# FTO is necessary for the induction of leptin resistance by high-fat feeding

**DOI:** 10.1016/j.molmet.2015.01.011

**Published:** 2015-02-07

**Authors:** Y.C. Loraine Tung, Pawan Gulati, Che-Hsiung Liu, Debra Rimmington, Rowena Dennis, Marcella Ma, Vladimir Saudek, Stephen O'Rahilly, Anthony P. Coll, Giles S.H. Yeo

**Affiliations:** 1 University of Cambridge Metabolic Research Laboratories and MRC Metabolic Diseases Unit, Wellcome-MRC Institute of Metabolic Science, Addenbrooke's Hospital, Cambridge, UK; 2 Department of Physiology, Development and Neuroscience, University of Cambridge, Cambridge, UK

**Keywords:** Fto, Leptin resistance, High-fat diet, NFкB, TRIP4, Hypothalamus, SOCS3, GWAS, Genome-wide association studies, SNPs, single nucleotide polymorphisms, FTO, *FaT mass and Obesity related*, HFD, high-fat diet, MEF, Mouse embryonic fibroblasts, Y2H, Yeast two-hybrid, Irx3, Iroquois Homeobox 3, ICV, intracerebroventricular injection, Ob-R, leptin receptor, SOCS3, suppressor of cytokine signalling, Tlr4, Toll-like receptor 4, PTPs, protein-tyrosine phosphatase, WAT, white adipose tissue

## Abstract

**Objective:**

Loss of function *FTO* mutations significantly impact body composition in humans and mice, with Fto-deficient mice reported to resist the development of obesity in response to a high-fat diet (HFD). We aimed to further explore the interactions between FTO and HFD and determine if FTO can influence the adverse metabolic consequence of HFD.

**Methods:**

We studied mice deficient in FTO in two well validated models of leptin resistance (HFD feeding and central palmitate injection) to determine how *Fto* genotype may influence the action of leptin. Using transcriptomic analysis of hypothalamic tissue to identify relevant pathways affected by the loss of *Fto*, we combined data from co-immunoprecipitation, yeast 2-hybrid and luciferase reporter assays to identify mechanisms through which *FTO* can influence the development of leptin resistant states.

**Results:**

Mice deficient in *Fto* significantly increased their fat mass in response to HFD. *Fto*^+/−^ and *Fto*^−/−^ mice remained sensitive to the anorexigenic effects of leptin, both after exposure to a HFD or after acute central application of palmitate. Genes encoding components of the NFкB signalling pathway were down-regulated in the hypothalami of *Fto*-deficient mice following a HFD. When this pathway was reactivated in *Fto*-deficient mice with a single low central dose of TNFα, the mice became less sensitive to the effect of leptin. We identified a transcriptional coactivator of NFкB, TRIP4, as a binding partner of FTO and a molecule that is required for TRIP4 dependent transactivation of NFкB.

**Conclusions:**

Our study demonstrates that, independent of body weight, Fto influences the metabolic outcomes of a HFD through alteration of hypothalamic NFкB signalling. This supports the notion that pharmacological modulation of FTO activity might have the potential for therapeutic benefit in improving leptin sensitivity, in a manner that is influenced by the nutritional environment.

## 1 Introduction

Genome-wide association studies (GWAS) have indicated that single nucleotide polymorphisms (SNPs) on chromosome 16 within the first intron of *FTO* (*FaT mass and Obesity related*) are robustly associated with human obesity [1]. These SNPs are also associated with obesity-related anthropometric traits such as fat mass, leptin levels and waist-to-hip ratio, as well as an increase in food intake and a reduction in satiety. More detailed analyses have determined that the increase in consumed energy in risk allele carriers appears due, at least in part, to an increased in preference for energy-dense food, especially those with a higher fat content [2,3]. FTO is a member of the ALKB family of nucleic acid demethylases and has a high affinity for 3-methylthymine (3-meT) in DNA [4] as well as 3-methyluracil (3-meU; [4,5]) and 6-methyladenosine (6-meA; [6]) in RNA.

The intronic SNPs associated with human obesity may influence adiposity through effects on the expression of other neighbouring genes including *IRX3* [7] and *RPGRIP1L* [8]. Whatever the explanation for the effects of intronic polymorphism on human adiposity, studies of humans and mice carrying genetic variants that functionally perturb FTO indicate that FTO itself is an important regulator of body size and composition, with loss of function mutations being associated with low body weight in humans [9] and mice [10,11,12] and transgenic overexpression resulting in obesity in mice [13]. *Fto* is highly expressed within the hypothalamus [1,4] and *Fto* mRNA levels in the arcuate nucleus are reduced by fasting [4,14,15] and increased by high-fat feeding [15,16]. Notably, *Fto*^+/−^ and *Fto*^−/−^ mice have been reported to be protected from the development of obesity on a high-fat diet (HFD) [10] while high-fat feeding exacerbates the obesity of mice overexpressing *Fto* [13].

The concept of “leptin resistance” has emerged to explain why obese animals, with high circulating leptin levels, do not appear to demonstrate the anorectic effects of leptin that are present in lean animals. High-fat feeding is a classical model whereby resistance to leptin can be induced in an experimental model. This can be replicated acutely by the central infusion of fatty acids, including palmitate [17]. More recently the notion has emerged that high-fat feeding and high central exposure to lipids can induce a state of inflammation in the hypothalamus and that this might be an important part of the induction of leptin resistance [18].

In this study, we provide further evidence that *Fto*^+/−^ and *Fto*^−/−^ mice remain sensitive to the anorectic effects of leptin despite exposure to either a HFD or the acute central administration of palmitate. We also identify a transcriptional co-activator that physically and functionally interacts with FTO to activate hypothalamic NFкB signalling pathways that have previously been implicated in the pathogenesis of leptin resistance.

## 2 Material and methods

### 2.1 Mice

*Fto* deficient mice were a generous gift from Prof. Roger Cox (MRC Harwell, Oxford) and were generated and genotyped as previously described [12]. All mice were maintained under controlled temperature (22 °C) and on a 12hr light, 12hr dark schedule (light on 07:00–19:00). Food and water were available *ad libitum* unless otherwise stated. All mice were weaned at 3 weeks of age onto a standard chow (SDS RM3, Essex, UK). All protocols were in accordance with the United Kingdom Home Office legislation (Animal (Scientific Procedure) Act 1986).

#### 2.1.1 Dietary intervention

Standard chow throughout all studies was SDS RM3 (Essex, UK). High fat diet used contains 60% of calories from fat, 20% from protein and 20% from Carbohydrate (D12492, Research Diet).

#### 2.1.2 Body composition analysis

Body composition was determined using dual-energy X-ray absorptiometry (DEXA, PIX-Imus2 Series Densimeters) under terminal anaesthetic (Dolethal, Vetoquinol UK Ltd).

#### 2.1.3 Glucose homeostasis

Oral glucose tolerance test (OGTT; 2 g glucose/kg) was performed in mice that were fasted for 6 h (beginning at 8AM) following 5 weeks on a HFD. Briefly, a bolus of 20% glucose was delivered into the stomach by a gavage needle and blood for measurement of glucose was taken by tail vein sampling at 0, 10, 20, 30, 60, 90 and 120 min and assayed with a glucometer (AlphaTRAK2, Abbott Animal Health).

### 2.2 Intracerebroventricular experiments

To allow ICV injection, male mice aged between 10 and 13 weeks underwent stereotaxic surgery to place an indwelling guide cannula into the lateral ventricle. Mice were anaesthetised with a mix of inhaled isofluorane and oxygen and a 26 gauge steel guide cannula (internal diameter 0.24 mm, outer diameter 0.46 mm, length 2 mm; Semat International, UK) was implanted into the right lateral ventricle using the following coordinates: 1.0 mm lateral from bregma, 0.5 mm posterior to bregma; 2.0 mm depth. The guide cannula was secured to the skull using quick-drying cyanoacrylate glue and dental cement (Associated Dental Products, Wiltshire, UK), and a dummy cannula was inserted. All animals received analgesia (Rimadyl, 5 mg/kg, Pfizer Animal Health, Kent, UK) and antibiotic (Teramycin LA, 60 mg/kg, Pfizer Animal Health, Kent, UK) before being returned to their home cage. Following surgery, mice were single housed throughout the study, food intake and body weight was measured daily. Injections were performed while mice were in their home cages and food returned 60 min after the last injection.

#### 2.2.1 Leptin responsiveness

8 week old male mice of each genotype were given either standard chow diet or HFD for four weeks, and had indwelling guide cannula placed as described above. Seven days post-surgery mice were reassessed and only those that had remained weight stable or put on weight were studied. After an overnight fast, mice received either 2 μl of PBS or recombinant murine leptin (500 ng in 2 μl; Amgen) *via* the cannula. Standard chow or high-fat diet was returned to the hopper and the amount consumed after 4- and 24-hr was measured.

#### 2.2.2 Palmitate injection

10–13 week old male mice maintained on standard chow were used. Mice were fasted overnight and 1 μl of each injectant or corresponding vehicle control was injected according to the respective experimental set-up (Figures 3 & 5). A crossover design was used such that each mouse served as its own control. Animals were allowed a washout period of at least 5 days between treatments and the order of treatments were re-randomized between experiments. Stock solution of 3.3 mM palmitate (Cat. 76119, Sigma) was prepared with 100% EtOH at 60 °C and further diluted with fatty acid-free BSA (Cat. A6003, Sigma) to give a final concentration of 66uM/μl. BSA or PBS Saline vehicle injectants were prepared equivalently, but without palmitate or recombinant mouse TNFα (aa 80–235; R & D System), respectively.

### 2.3 Immunohistochemistry

Adult male mice were deeply anesthetised with sodium pentobarbital and were transcardially perfused with PBS followed by ice-cold 10% formalin. After perfusion, brains were removed and post-fixed in 15% sucrose formalin then transferred to 30% (wt/vol) sucrose in PBS overnight. Perfused brains were sectioned at 30 μm thickness on a microtome in a one-in-four coronal series through the hypothalamus. Phosphorylated STAT3 immunohistochemistry was performed as previously described [19] using a rabbit anti-pSTAT3 antibody (Cat. No.9131; Cell Signaling Technology). Briefly, tissues were treated sequentially with 1% NaOH and 1% H_2_O_2_ for 20 min, 0.3% glycine for 10 min and 0.03% SDS for 10 min. Sections were then blocked with normal goat serum and incubated with anti-pSTAT3 antibody (1:2000) overnight at 4 °C. The next day, sections were incubated with biotinylated goat anti-rabbit antibody (1:2000, Dako) followed by avidin-biotin complex solution (Elite ABC Kit, Vector Laboratories) and brown precipitate development by diaminobenzidine solution (ImmPACT^TM^DAB, Vector Laboratories). pSTAT3-positive cells were clearly defined by visual inspection in the arcuate nucleus of hypothalami from mice given central fatty acid pre-treatment followed by leptin. pSTAT3 positive cells were counted bilaterally under a light microscope from matched sections ranging from bregma −1.22 to −2.30 mm (Paxinos Mouse Brain Atlas). The investigator was blinded to the treatment group and expressed counts as the total number of pSTAT3 positive cells per section. Images were acquired with an Olympus BX41 microscope equipped with the Colorview soft imaging system.

### 2.4 Quantitative real-time PCR

One hour after leptin injection, mice were euthanized by terminal anaesthesia (Dolethal, Vetoquinol UK Ltd) and hypothalami rapidly removed and flash frozen in liquid nitrogen and stored at −80 °C until further analysed.

Total RNA from the tissue of interest was purified with TRIsure (Bioline) using a FastPrep 24 homogeniser (MP Biomedicals) according to the manufacturer's instructions. The RNA was reverse transcribed and amplified using TaqMan Universal PCR Master Mix with TaqMan Assay on demand kit (Applied Biosystems). Quantitative PCR reactions were performed in duplicate and relative expression was determined for target mRNA and adjusted for total mRNA content by hypoxanthine-phosphoribosyl transferase (*Hprt*), TATA – binding protein (*Tbp*) and β2 – macroglobulin (*B2M*). Quantitative PCR statistical analysis was performed using Microsoft Excel. P-value was calculated using a two-tailed distribution unpaired Student's t-test and data expressed as mean ± SEM.

For the NFκB signalling pathway profiler PCR array (Mouse NFκB Signalling Pathway, Qiagen), total RNA was reverse transcribed according to the manufacturer's instructions using 400 ng starting material. Arithmetic mean of *B2M*, Heat shock protein 90 KDa alpha class B member 1 (*Hsp90ab1*) and B-glucuronidase (*Gusb*) expression level was used as loading controls.

### 2.5 Flag-tag pull down and endogenous immunoprecipitations

Mouse embryonic fibroblasts (MEF) were obtained from *Fto* WT and null mice. MEFs and HEK 293 cells were maintained in Dulbecco's Modified Minimal Essential Medium (DMEM5796, Sigma) supplemented with 10% foetal bovine serum at 37 °C in the presence of 5% CO_2_. For over expression studies, transient transfections were performed using a CalPhos kit (for HEKs 293) from ClonTech and Neon System (for MEFs) from Invitrogen according to the manufacturer's protocol. Flag-tag, FTO and TRIP4 IPs were performed as described previously [20]. After the IPs, immunocomplexes were eluted using glycine buffer (pH 3.0) at 4 °C for 30 min. After the elution remaining beads and eluate were resuspended in 1x sample buffer for Western blot analysis.

### 2.6 Luciferase assays

For the luciferase study, assays were performed with the Cignal NFκB Reporter Assay (Promega). Briefly, MEF cells derived from Fto null mice were transfected with the Cignal reporters which is a mixture of an inducible NFκB- responsive firefly luciferase construct and a constitutively expressing renilla luciferase construct, together with various combinations of expression vectors under investigation. “Empty” vector controls expresses GFP and was also used to monitor consistency in transfection efficiency. All transfections were performed with the Neon transfection system (Invitrogen), following the manufacturer's instructions, and enzymatic activities were measured using a luminometer (DLReady; Berthold Technologies) performed with the Dual-luciferase reporter system (Promega). Briefly, firefly luciferase reagent (LARII) was added to the test sample followed by addition of equal amounts of renilla luciferase reagent and firefly quenching reagent (Stop & Glo). The data are represented as the ratio of firefly to renilla luciferase activity and is the mean of at least three separate experiments performed in triplicate.

### 2.7 Testing of the direct *in vivo* interaction between FTO and TRIP4 in the Y2H system

Yeast two-hybrid is a widely used method for studying protein–protein interactions *in vivo*, so we used the commercially available Matchmaker Gold Yeast Two-Hybrid System (Clontech). In these assays, FTO is expressed as a fusion to the GAL4 DNA-binding domain (BD), while TRIP4 is expressed as fusion to the GAL4 activation domain (AD). When FTO and TRIP4 fusion proteins interact, the DNA-BD and AD are brought into close proximity, thus activating transcription of four independent reporter genes (AUR1-C, ADE2, HIS3 and MEL1). The combination of antibiotic, nutritional and colorimetric reporters means false positive are reduced to a minimal and allow for “grading” of the binding strength to be determined, with activation of all four reporters suggesting the highest binding strength. Nine truncated fragments of the human FTO functional domains were tested for their ability to bind to the full length TRIP4 directly.

All of the procedures were performed according to the manufacturer's protocol (Clontech). Briefly, full length or truncated constructs of FTO were cloned into the vector pGBKT7 and were used to transfect the yeast strain AH107 and the pGADT7-TRIP4 recombinant plasmids were transfected into the yeast strand Y180. The yeast strains Y187-pGADT7-TRIP4 and AH109-pGBKT7-FTO were tested for autonomous activation, and none of the constructs showed any self-activation. The transfected strains were used for the yeast two-hybrid mating assays testing the binding domains for interaction between FTO and TRIP4. The “mated” transformants were cultured in medium for 20hr and then plated onto the appropriate selective medium. The strength of the interaction was confirmed by colonies growing on the selective medium, with the highest binding denoted as “++++” assigned to colonies growing on fully selective medium; while “−“ was assigned to background colony growth. All data presented are repeats of at least 3 separate experiments and most of the interactions were done with TRIP4 cloned into the vector pGADT7 (containing AD) and FTO cloned into vector pGBKT7 (containing BD); but for a selection of interaction studies, the reverse were also assayed to eliminate potential bias and the obtained results were comparable in either cloning direction.

### 2.8 Statistical analysis

All data are presented as mean ± SEM and have been analysed using Microsoft Excel and GraphPad Prism 5. Single comparisons were made using one- or two-tail Student's t-test as specified in the respective figure legends. Multiple comparisons were tested with ANOVA and adjusted with Bonferroni's post-test. Difference among groups were considered significant for p-values of <0.05.

## 3 Results

### 3.1 Influence of Fto status on the change of body composition in response to a HFD

**Figure 1:**
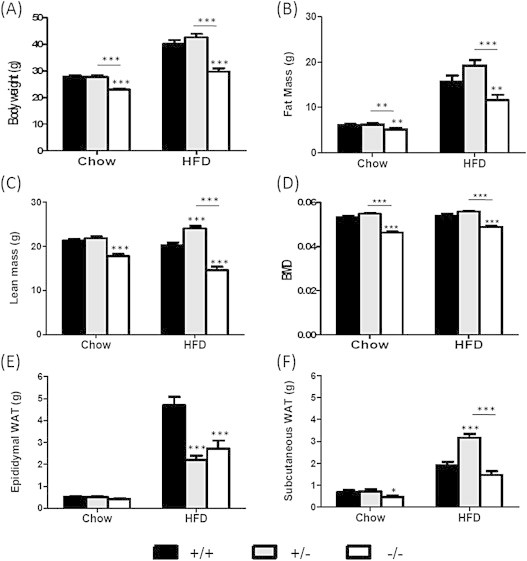
**Effect of exposure to a high-fat diet in Fto-deficient mice**. Effect of Fto-deficiency on anthropometric measurements of 13 week old male mice either on standard chow or 5 weeks of 60% HFD. (A) Body weight; (B) total fat mass; (C) total lean mass and (D) Bone mineral density as measured by dual-energy X-ray absorptiometry (DEXA). (E) Weights of dissected epididymal white adipose tissue and (F) subcutaneous white adipose tissue. Black bars denote *Fto*^+/+^ (n = 17 chow, n = 13 HFD); grey bars denote *Fto*^+/−^ (n = 20 chow, n = 13 HFD); white bars denote *Fto*^−/−^ (n = 13 chow, n = 10 HFD). *p < 0.05; **p < 0.01; ***p < 0.001 following two-tailed t-test.

We were intrigued by the previous reports that Fto-deficient and haplo-insufficient mice did not develop obesity in response to a high-fat diet [10] and initially set out to see if this phenomenon was readily replicable. Eight week old WT, *Fto*^+/−^ and *Fto*^−/−^ mice were given a 60% HFD for five weeks and their body composition subsequently compared to age-matched mice of the same genotypes that had been continuously provided with regular chow for the same time period. As previously reported, on regular chow *Fto*^−/−^ mice had a significant reduction in total body weight (Figure 1A), fat mass (Figure 1B) and lean mass (Figure 1C), as well as bone mineral density (Figure 1D), when compared to WT littermates.

As expected, after five weeks on HFD diet, WT mice weighed 44% more than mice fed chow (40.1 ± 1.5 g vs 27.9 ± 0.6 g; p < 0.001) (Figure 1A). However, in contrast to previous reports both Fto^+/−^ and Fto^−/−^ significantly increased their body mass on HFD. The response of *Fto*^+/−^ mice was not significantly different from that of WT (42.6 ± 1.3 g on HFD vs 27.7 ± 0.7 g on chow, a 53% increase; p < 0.001), while *Fto*^−/−^ mice increased their weight by 29% (29.7 ± 1.2 g on HFD vs 23 ± 0.5 g on chow; p < 0.001).

**Supplemental Figure 1:**
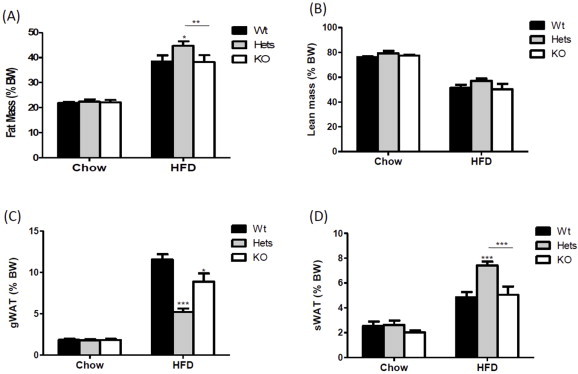
Effect of Fto-deficiency on anthropometric measurements of 13 weeks old male mice either on standard chow or 5 weeks of 60% HFD expressed as percentage of body weight. (A) Total fat mass; (B) total lean mass measured by dual-energy X-ray absorptiometry (DEXA). (C) Weights of dissected epididymal white adipose tissue and (D) subcutaneous weight adipose tissue. Black bars denote *Fto*^+/+^ (n = 17 chow, n = 13 HFD); grey bars denote *Fto*^+/−^ (n = 20 chow, n = 13 HFD); white bars denote *Fto*^−/−^ (n = 13 chow, n = 10 HFD). *p < 0.05; **p < 0.01; ***p < 0.001 following two-tailed t-test.

**Supplemental Figure 2:**
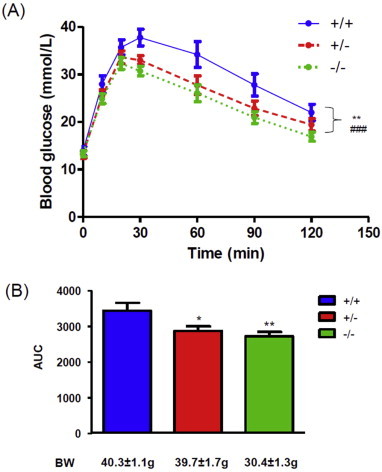
The ability of WT and Fto-deficient male mice on chow and a HFD to reduce food intake in response to an intracerebroventricular (icv) injection of leptin. A single injection of 500 ng of leptin was administered centrally and food intake was measured 4 h after food was returned to the hopper. (A) Leptin administration decreased food intake significantly in all mice on standard chow (*Fto*^+/+^ n = 8, *Fto*^+/−^ n = 11, *Fto*^−/−^ n = 6). (B) Leptin-induced anorexia is absent in WT mice with 5 weeks of high-fat feeding but retained in Fto-deficient mice (*Fto*^+/+^ n = 10, *Fto*^+/−^ n = 15, *Fto*^−/−^ n = 7). Data are plotted as grams of food consumed for the 4 h duration post saline or leptin injection. *p < 0.05; **p < 0.01; ***p < 0.001; NS = not significant following one-tailed t-test.

We went on to analyse body composition in each genotype and determined that the increase in body mass in each genotype was primarily driven by a significant increase in fat mass (Figure 1B & Supplemental Figure 1A), indicating that Fto loss does not protect from HFD diet induced obesity. Despite a lowered total absolute fat mass in the *Fto*^−/−^ mice compared to WT and *Fto*^+/−^ mice, there was no difference in serum leptin levels between the three genotypes either on regular chow (WT *vs Fto*^+/−^ *vs Fto*^−/−^; 2.37 ± 0.34 ng/ml *vs* 3.24 ± 0.51 ng/ml *vs* 2.33 ± 0.59 ng/ml, respectively, n.s.) or after a HFD (WT *vs Fto*^+/−^ *vs Fto*^−/−^; 25.11 ± 2.7 ng/ml *vs* 27.5 ± 5.8 ng/ml *vs* 31.6 ± 8.6 ng/ml, respectively, n.s.). Intriguingly, *Fto* genotype appeared to have some influence on the distribution of body fat in response to a HFD. WT mice given a HFD gained more visceral epididymal fat (4.7 ± 0.38 g) than subcutaneous fat (1.9 ± 0.15 g; p < 0.001) (Figure 1E, F & Supplemental Figure 1C, 1D). In contrast, HFD fed *Fto*^+/−^ mice with similar absolute fat mass as WT (Figure 1B), had more subcutaneous than epididymal fat (3.2 ± 0.17 g vs. 2.2 ± 0.17 g, respectively; p < 0.001; Figure 1E,F), and displayed an improved glucose tolerance as compared to WT mice on HFD (Supplemental Figure 2).

### 3.2 Despite a significant increase in fat mass, Fto-deficient mice remain leptin sensitive on a HFD

**Supplemental Figure 3:**
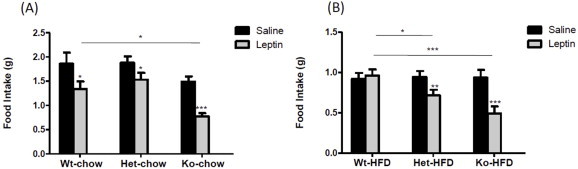
Glucose tolerance is improved in Fto deficient mice compared to *Fto*^+/+^ mice following 5 weeks of 60% HFD. (A) Oral glucose tolerance test (OGTT) was performed in mice fasted for six hours and blood samples were obtained for glucose measurement at indicated time points for *Fto*^+/+^ (n = 10, blue); *Fto*^+/−^ (n = 11, red) and *Fto*^−/−^ (n = 10, green). **p < 0.01 (*Fto*^+/+^ vs. *Fto*^+/−^), ###p < 0.001 (*Fto*^+/+^ vs. *Fto*^−/−^) analysed by repeated measures ANOVA followed by the Bonferroni multiple comparison test. (B) Area under the curve [26] for blood glucose during OGTT. *p < 0.05; **p < 0.01 following two-tailed t-test.

**Figure 2:**
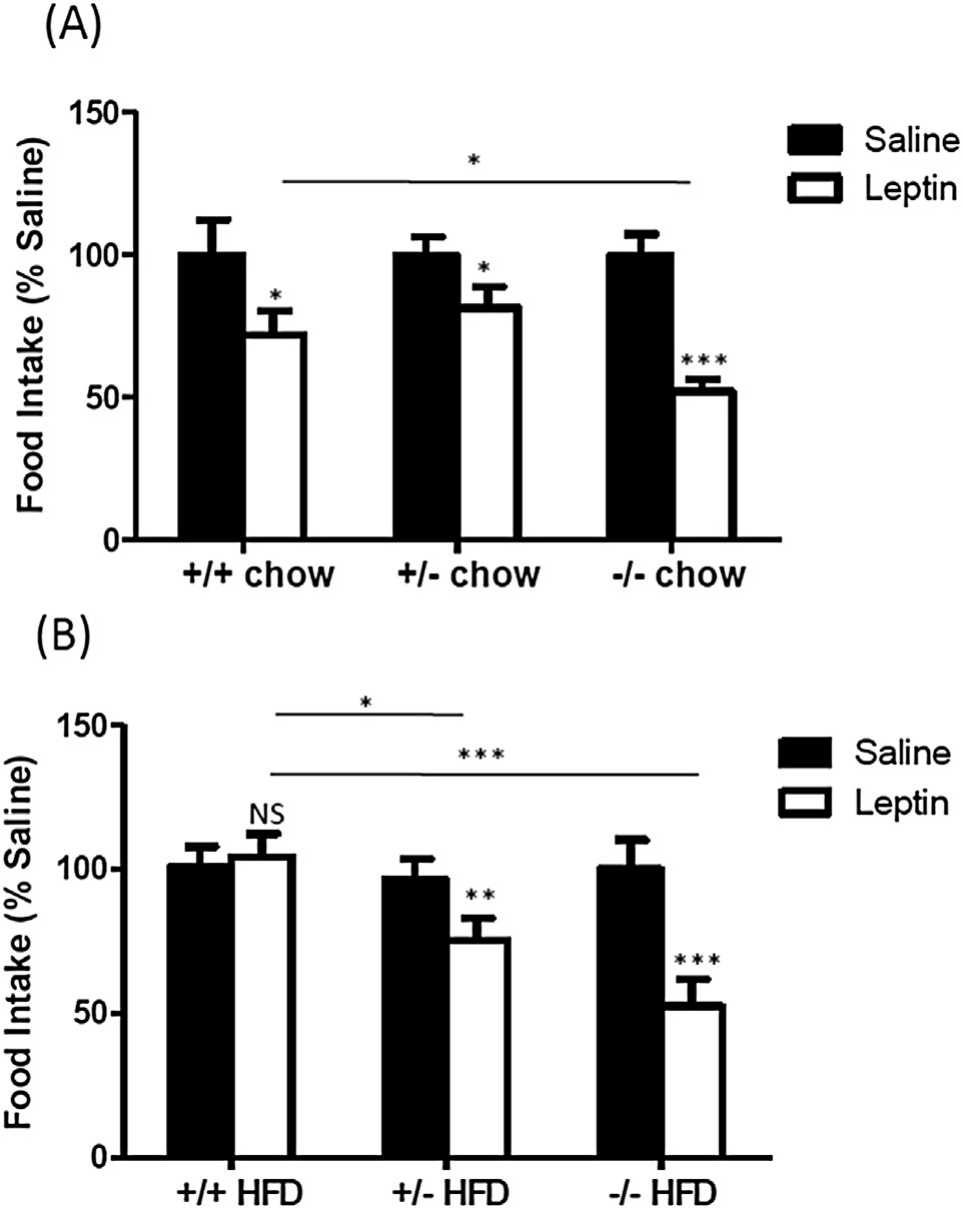
**Measurement of leptin sensitivity in Fto-deficient mice exposed to a high-fat diet**. The ability of WT and Fto-deficient male mice on chow and a HFD to reduce food intake in response to an intracerebroventricular (icv) injection of leptin. A single injection of 500 ng of leptin was administered centrally and food intake was measured 4 h after food was returned to the hopper. (A) Leptin administration decreased food intake significantly in all mice on standard chow (*Fto*^+/+^ n = 8, *Fto*^+/−^ n = 11, *Fto*^−/−^ n = 6). (B) Leptin-induced anorexia is absent in WT mice with 5 weeks of high-fat feeding but retained in Fto-deficient mice (*Fto*^+/+^ n = 10, *Fto*^+/−^ n = 15, *Fto*^−/−^ n = 7). Data are plotted as percentage of values obtained, with food intake in the saline-treated control group in each genotype set as 100%. *p < 0.05; **p < 0.01; ***p < 0.001; NS = not significant following one-tailed t-test.

In light of these findings, we went on to test the sensitivity of all three groups to central leptin administration. On a chow diet, all three genotypes were responsive to the anorexigenic effects of leptin with *Fto*^−/−^ mice demonstrating the most robust response (50% drop in 4hr food intake; p < 0.001) (Figure 2A & Supplemental Figure 3A). As expected, after five weeks on a HFD, WT mice had developed leptin resistance as judged by a failure to reduce food intake in response to the same dose of leptin that brought about a decrease in food intake in chow fed animals. Intriguingly, obese HFD fed Fto-deficient mice remained sensitive to leptin in a gene-dosage manner, with *Fto*^+/−^ mice reducing their food intake by 20% (p < 0.01) and *Fto*^−/−^ mice by 50% (p < 0.001) (Figure 2B & Supplemental Figure 3B).

### 3.3 Fto-deficient mice remain sensitive to leptin after acute central exposure to palmitate

**Figure 3:**
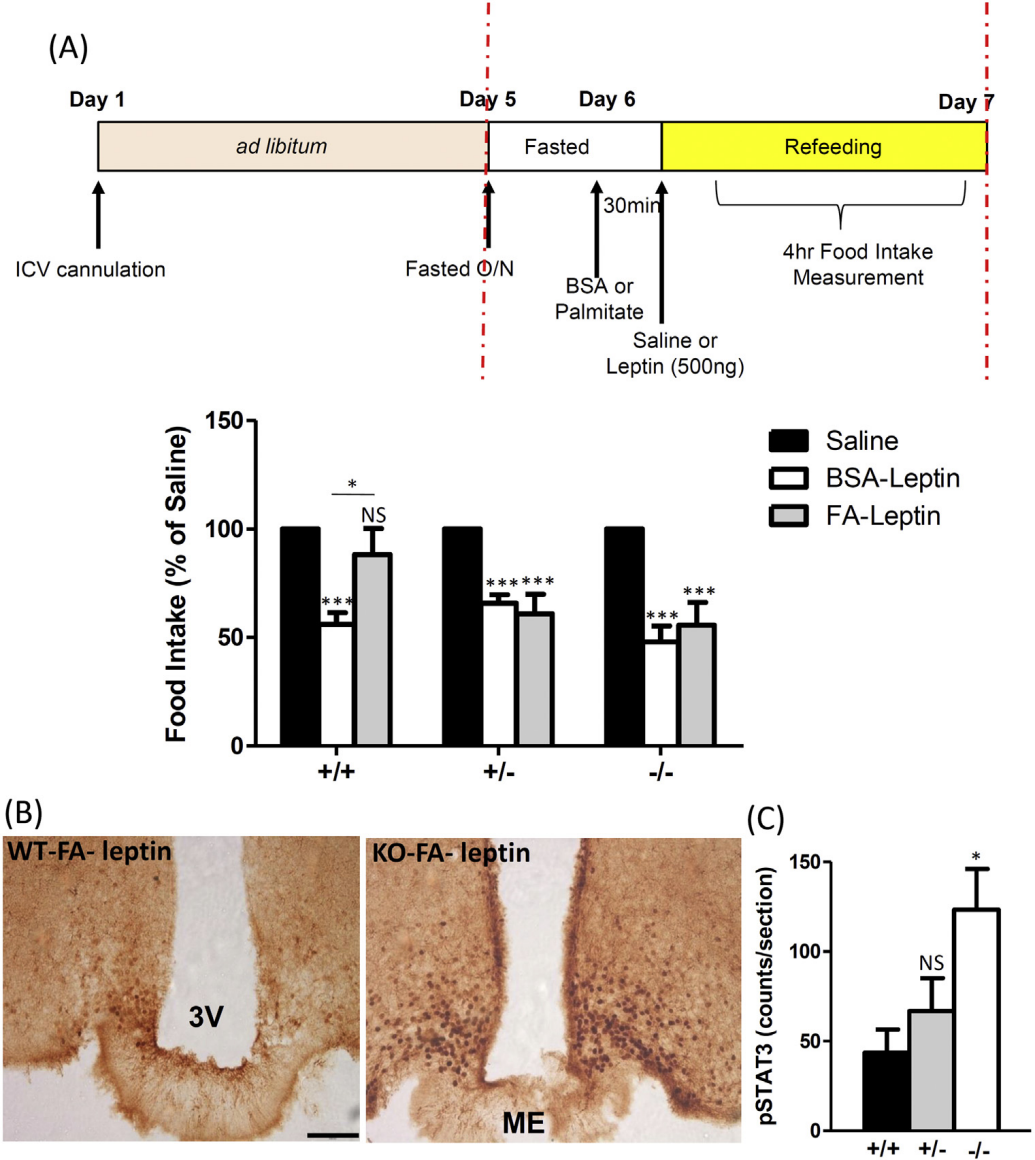
**Measurement of leptin-sensitivity in Fto-deficient mice injected centrally with palmitate**. (A) Palmitate was administered 30 min prior to leptin to test for its ability to block leptin-induced anorexia. Mice on standard chow from each genotype were fasted overnight prior to injection of palmitate or BSA control and followed by leptin, as illustrated in the schematic of our experimental strategy (*Fto*^+/+^ n = 10, *Fto*^+/−^ n = 9, *Fto*^−/−^ n = 8). Data are plotted as a percentage of the value obtained with saline treatment. (B) Effect of palmitate pre-treatment on leptin induced p-STAT3 immunostaining in representative coronal sections from *Fto*^+/+^ and *Fto*^+/−^ mice. (C) Number of p-STAT3 immunoreactive arcuate nucleus neurons with n = 3 mice per group. 3V = third ventricle; ME = median eminence. Bar = 100um. Data are presented as mean ± SEM. *, p < 0.05; **, p < 0.01; ***, p < 0.001; NS = not significant following two-tailed t-test.

Given that Fto is highly expressed within the hypothalamus, we hypothesised that effects seen in Fto deficient mice were a direct result of loss of central expression of Fto. To investigate this further, we utilised a second, well characterised model of acute leptin resistance, that of central injection of palmitate [17] (Figure 3A). As expected, pre-treatment with palmitate prior to leptin injection induced almost complete central leptin resistance in WT mice. However, *Fto*^+/−^ and *Fto*^−/−^ mice remained fully sensitive to leptin even after palmitate pre-treatment, with both groups reducing food intake by approximately 40% (p < 0.001) in response to leptin (Figure 3A). This recapitulates the phenotype seen after a 5 week exposure to HFD and argues that the mechanisms underlying the maintained sensitivity to leptin in *Fto*^+/−^ and *Fto*^−/−^ mice are directly centrally mediated. In keeping with this retained sensitivity to leptin after palmitate treatment, there was a significant increase in leptin-induced phosphorylation of STAT3 in the hypothalamic ARC of *Fto*^−/−^ compared to WT mice (p < 0.05) (Figure 3B,C). We did see an intermediate phenotype in the *Fto*^+/−^ mice, although this was not statistically significant (Figure 3C).

### 3.4 NFκB signalling pathway is down-regulated in Fto-deficient mice on HFD

**Supplemental Figure 4:**
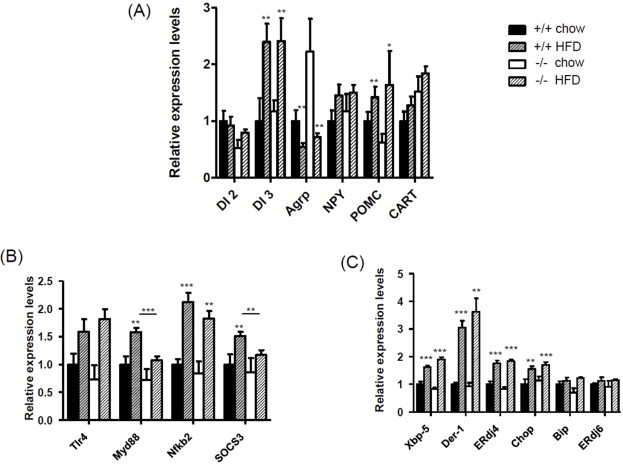
Relative mRNA levels for (A) classical hypothalamic neuropeptide; (B) NFκB pathway related genes and (C) markers of endoplasmic reticulum stress were determined by quantitative RT-PCR in the hypothalamus of *Fto*^+/+^ and *Fto*^−/−^ mice fed either a normal chow or exposed to 5 weeks of high-fat diet from 8 weeks of age. Data are means ± SEM. *, p < 0.05; **, p < 0.01; ***, p < 0.001 following two-tailed t-test. Each mRNA was normalised to housekeeping *Tbp* mRNA in the same sample.

We looked next to determine the underlying molecular mechanism that allow Fto-deficient mice to remain leptin sensitive on a HFD by examining gene expression within the hypothalamus, a region critically involved in appetite regulation and one in which Fto is highly expressed (Supplemental Figure 9). Analysis of canonical leptin responsive genes within the ARC showed no differences in expression of *Agrp*, *Npy*, *Pomc*, *Cart* or deiodinase type II (DI2) & type III (DI3) in response to a HFD between WT and *Fto*^−/−^ mice (Supplemental Figure 4A).

**Figure 4:**
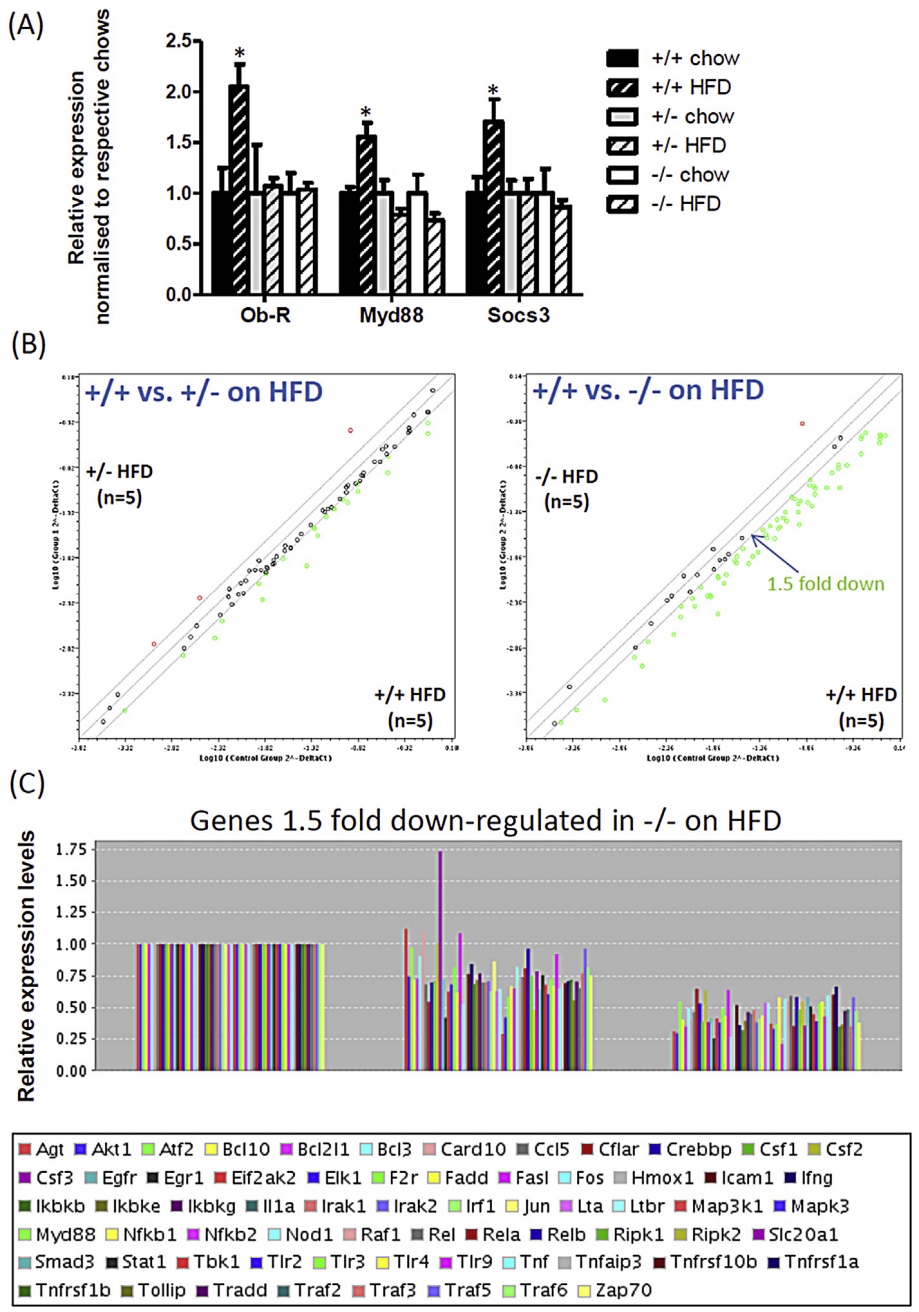
**Hypothalamic gene expression in Fto-deficient mice exposed to a HFD**. (A) Relative mRNA levels for *leptin receptor (Ob-R)*, *Myd88* and suppressor of cytokine signalling (*SOCS3*) were determined by quantitative RT-PCR in the hypothalamus of mice from each genotype fed normal chow versus mice exposed to 5 weeks of high-fat diet. Data are means ± SEM. * = p < 0.05 following two-tailed t-test. n = 9–15 mice per group. Each mRNA was normalised to *Hprt* mRNA in the same sample. (B) Scatter plot of the expression profile of 84 genes encoding components of the NFκB pathway. Each dot represents a gene and is obtained by plotting the log of the average normalised signal intensity of genes in the HFD state against the chow fed state. Green dots represent genes that are down-regulated with HFD and the red dots are those that are up-regulated during a HFD. (C) Bar plot of genes encoding components of the NFκB pathway that are 1.5 fold down-regulated in *Fto*^−/−^ mice.

We turned next to the pathways downstream of the leptin receptor. Leptin signalling *via* STAT3 induces the expression of *SOCS3* mRNA in the hypothalamus, which inhibits the phosphorylation and activation of JAK2. Over-activation of SOCS3 is a proposed mechanism for leptin resistance; particularly in diet induced obese states. We found a significant reduction in *Socs3* expression in the hypothalami of *Fto* deficient mice on a HFD as compared to WT mice (Figure 4A & Supplemental Figure 4B). In addition to playing a role in leptin signalling, SOCS3 is also downstream of NFкB signalling, a pathway known to be up-regulated within the medial-basal hypothalamus in response to a HFD. We measured expression of Myd88, a key adapter required for NFкB activation, and found that it too exhibited a significant reduced response in *Fto* deficient mice on a HFD as compared to WT mice (Figure 4A & Supplemental Figure 4B).

**Supplemental Figure 5:**
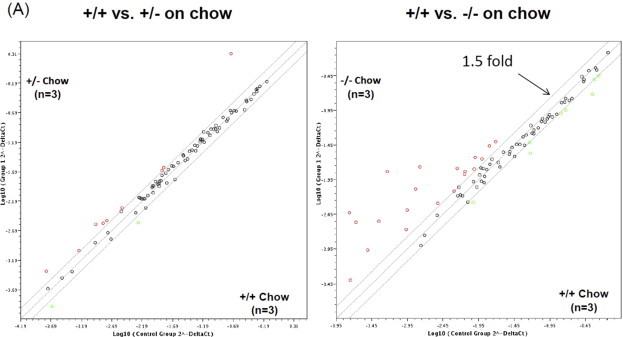
(A) Scatter plot of the expression profile of 84 genes encoding components of the NFκB pathway comparing *Fto*^+/+^ and Fto-deficient mice (*Fto*^+/−^ left panel or *Fto*^−/−^ right panel) fed on standard chow. Each dot represents a gene and red dots are those that are up-regulated in Fto-deficient mice while green dots represent genes that are down-regulated with Fto-deficiency. (B) Gene expression profile of components of the NFκB pathway comparing *Fto*^+/+^ on standard chow versus *Fto*^+/+^ mice exposed to 5 weeks of high-fat diet. (Left panel) Data presented as scatter plot obtained by plotting the log of the average normalised signal intensity of genes in the HFD state against the chow fed state. (Right panel) bar graph presented genes regulated by at least 1.5-fold following a high-fat feeding.

**Supplemental Figure 6:**
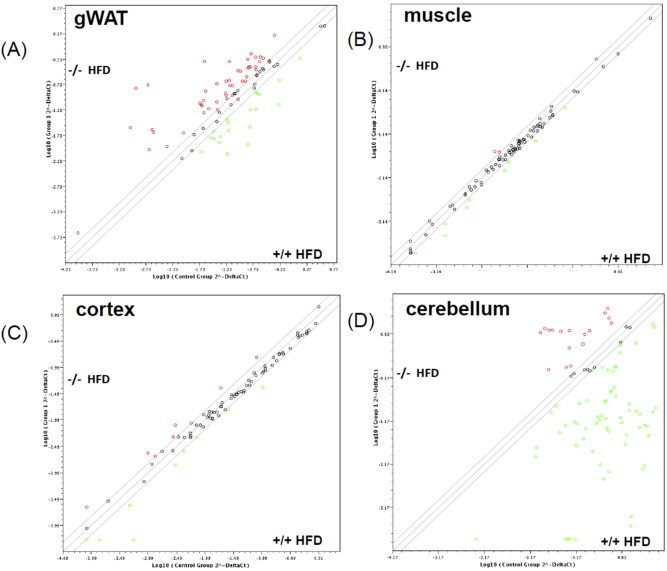
Scatter plot of the (A) epididymal white adipose tissue (gWAT); (B) muscle; (C) cortex or (D) cerebellum expression profile of 84 genes encoding components of the NFκB pathway. Each dot represents a gene and is obtained by plotting the log of the average normalised signal intensity of genes in the *Fto*^−/−^ mice against the *Fto*^+/+^ mice on HFD state. Green dots represent genes that are down-regulated in *Fto*^−/−^ mice and the red dots are those that are up-regulated in *Fto*^−/−^ mice.

These findings prompted us to examine the NFкB pathway more broadly and using a mouse NFкB signalling pathway profiler PCR array, we measured the gene expression profile of 84 components of NFкB signalling within the hypothalami of WT, *Fto*^+/−^ and *Fto*^−/−^ mice on chow and HFD. The array included genes that encode members of the NFкB, IkB families, NFкB-responsive genes, extracellular ligands and receptors that activate the pathway, as well as downstream kinases and transcription factors. We found no difference in expression pattern of NFкB pathway genes in the three different genotypes on a chow diet (Supplemental Figure 5A), while, as expected, there was up-regulation of 60 NFкB pathway genes in the hypothalami of WT mice exposed to a HFD (Supplemental Figure 5B & Supplemental Table 1). However, in Fto*-*deficient mice on HFD, we found a co-ordinate down-regulation of the NFкB pathway in a gene dosage dependent fashion, with *Fto*^−/−^ mice displaying a greater effect than that seen in *Fto*^+/−^ mice (Figure 4B, C & Supplemental Table 2). This co-ordinated down-regulation of NFкB pathway gene expression was specific to the hypothalamus and was not seen in other regions of the brain such as the cortex and cerebellum, or in peripheral tissues such as epididymal fat and skeletal muscle (Supplemental Figure 6).

In addition to the NFкB pathway, we also examined expression of a number of key markers of endoplasmic reticulum stress (ER stress), another cellular process that can cause inflammation during HFD feeding and has been suggested to lie downstream of Myd88 signalling [21]. However, no differences in expression were detected in several of the markers of ER stress activation [22] between *Fto*^−/−^ and WT mice (Supplemental Figure 4C).

### 3.5 TNFα activation of the NFкB pathway in Fto-deficient mice restores palmitate induced leptin resistance

**Figure 5:**
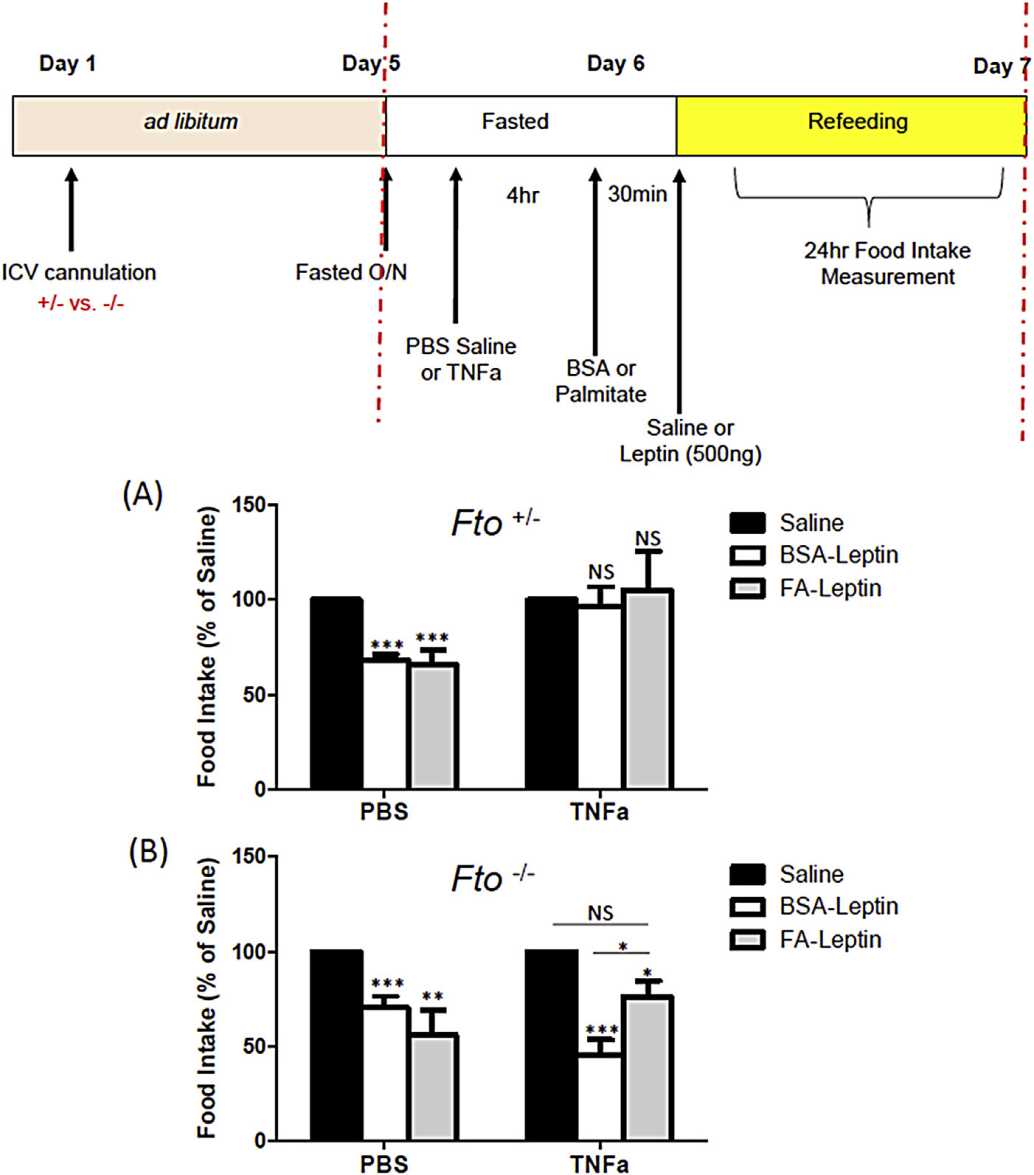
**Effects of central TNFα injection on palmitate induced leptin resistance**. To test the ability of TNFα upregulation of NFκB pathway gene expression to induce leptin resistance in palmitate treated Fto-deficient mice, TNFα or PBS control was injected centrally, four hours prior to palmitate and then leptin administration. Overnight fasted (A) *Fto*^+/−^ mice (n = 7 with PBS; n = 10 with TNFα) or (B) *Fto*^−/−^ mice (n = 7 with PBS; n = 8 with TNFα) on standard chow were treated as illustrated in the schematic of our experimental strategy. Data are presented as mean ± SEM and are plotted as a percentage of the value obtained with saline treatment. *p < 0.05; **p < 0.01; ***p < 0.001; NS = not significant following two-tailed t-test.

To establish if down-regulation of the hypothalamic NFкB pathway was causally related to the demonstrated leptin sensitivity, we used centrally administered TNFα to reactivate the pathway in Fto-deficient mice prior to fatty acid administration (Figure 5).

**Supplemental Figure 7:**
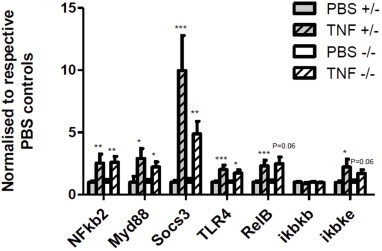
To test the ability of TNFα upregulation of NFκB pathway gene expression, hypothalami of *Fto*^+/−^ (n = 14 with PBS; n = 12 with TNFα) and *Fto*^−/−^ (n = 7 with PBS; n = 13 with TNFα) were obtained four hours after central injection of PBS saline or TNFα. Data are presented as mean ± SEM and are plotted as a ratio of the value obtained with respective saline treatment. *, p < 0.05; **, p < 0.01; ***, p < 0.001 following two-tailed t-test.

Central TNFα treatment has previously been shown to activate the NFкB pathway in the hypothalamus [23]. A dose of TNFα (10 pg) was used that had no effect on 24hr food intake (saline *vs* TNFα, *Fto*^+/−^ 7.7 ± 0.4 g *vs* 6.2 ± 0.7 g, N.S; *Fto*^−/−^ 7.1 ± 0.4 g *vs* 7.9 ± 0.3 g, n.s.) but up-regulated *Nfkb2, Myd88, Tlr4* and *Socs3* expression in the hypothalami of *Fto*^+/−^and *Fto*^−/−^ mice (Supplemental Figure 7). In contrast to the PBS control treatment (Figure 5A,B), TNFα pre-treatment resulted in sensitization of the *Fto*^−/−^ mice to palmitate, leading to a blunted response to leptin (Figure 5B), similar to that seen in the WT mice, where palmitate prior to leptin injection induced central leptin resistance (Figure 3A). In *Fto*^+/−^ mice, where NFкB pathway gene expression was modestly down-regulated (as compared to *Fto*^−/−^) (Figure 4B; 4C; Supplemental Table 2), treatment with TNFα alone was sufficient to induce leptin resistance without palmitate treatment (Figure 5A). Thus our data suggest that Fto influences the ability of a HFD to induce central leptin resistance through alteration of hypothalamic NFкB signalling.

### 3.6 FTO binds to TRIP4 and is necessary for TRIP4 dependent transactivation of NFкB

**Supplemental Figure 8:**
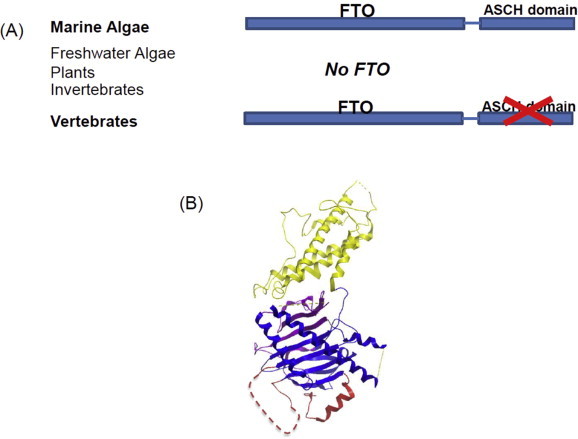
(A) FTO homologues exist in vertebrate and in marine algae but only in the algae it has an additional ASCH domain at the C’-terminus. (B) FTO binds to TRIP4 through two surface mobile epitopes (red) exposed at one side of FTO as visualised here in the 3-dimentional structure. Dashed lines indicate mobile loops invisible in the crystal structure and the structure is coloured according to structural units as in Figure 6C.

**Supplemental Figure 9:**
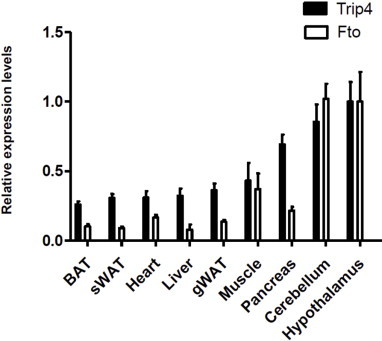
Relative mRNA levels for Trip4 (black bars) and Fto (white bars) were determined by quantitative RT-PCR in a tissue panel of WT mice fed standard chow. n = 5–6 for each tissue. Each mRNA was normalised too housekeeping gene *B2M* mRNA and data presented as a ratio to the expression in the hypothalamus, the tissue with highest expression of both *Trip4* and *Fto*.

We looked next for interacting partners to determine a possible mechanistic link between FTO and NFкB signalling. Based on bioinformatic analysis of FTO's phylogenetic evolution, we focused on a family of proteins that contained ASCH domains. Although FTO homologues exist in vertebrates and in marine algae, FTO of several algae has an additional C-terminal ASCH domain (Supplemental Figure 8A). It is a common observation that domains that are components of one polypeptide in some species and separated in different chains in other species enter the same functional pathway, and frequently physically interact [24]. There are only three ASCH domain-containing proteins in the mammalian genome, one of which is Thyroid receptor interacting protein 4 (TRIP4), a known transcriptional coactivator of NFкB [25]. *Trip4* is highly expressed in the CNS, particularly the hypothalamus (Supplemental Figure 9).

**Figure 6:**
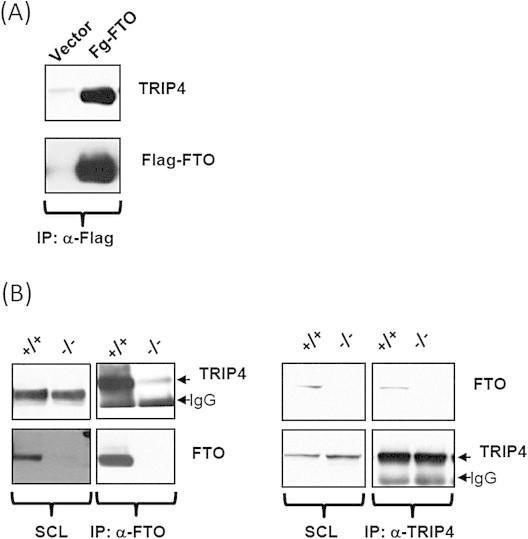

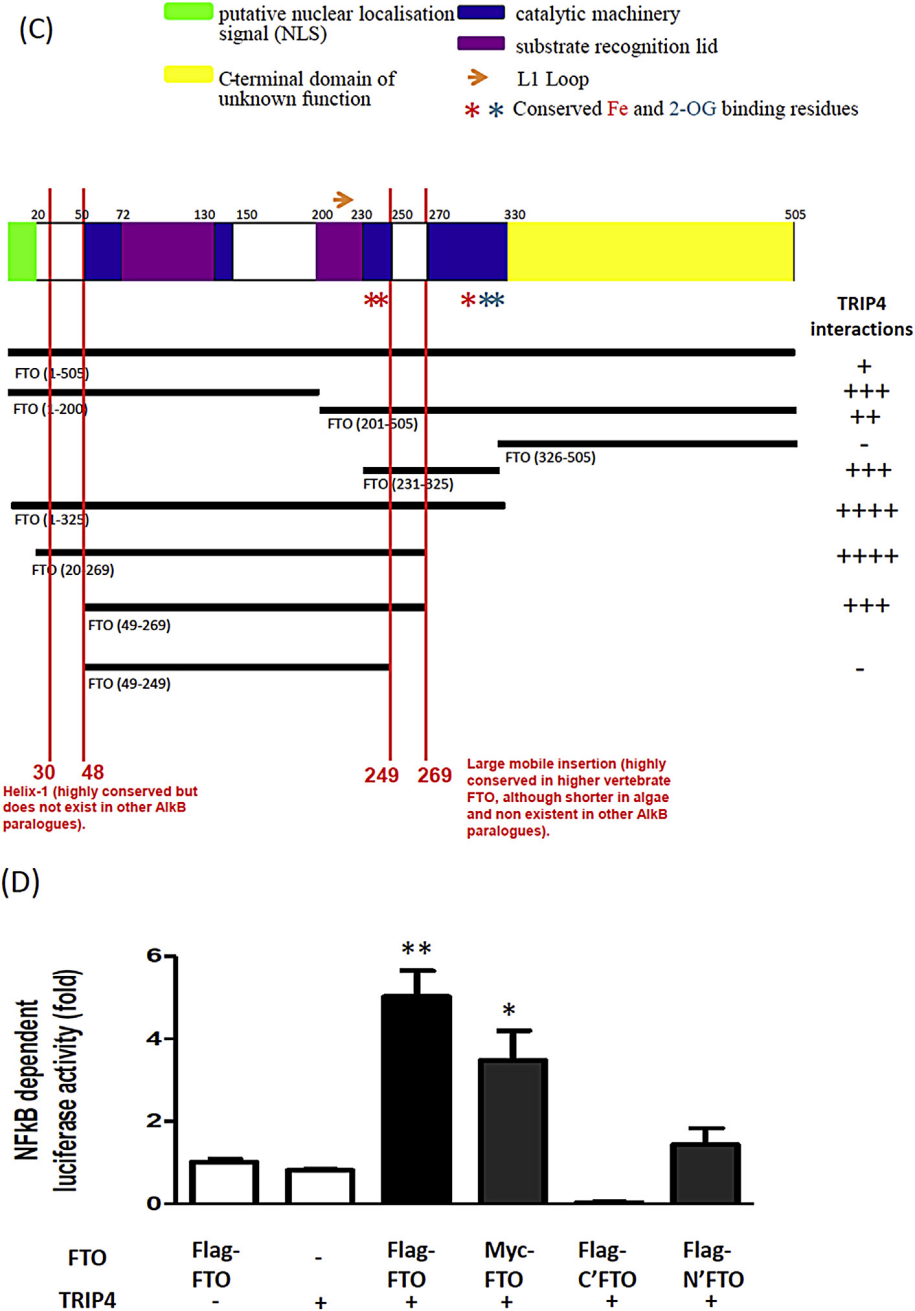
**FTO interacts with TRIP4, a transcriptional coactivator of NFκB**. (A) HEK293 cells were transiently transfected with vector alone or Flag- FTO. Cells were harvested after 48 h and resulting cell extracts were subjected to Flag immunoprecipitation using antibody against Flag protein. Flag immunoprecipitates were then eluted from the beads and analysed further by western blotting. (B) MEFs derived from wild type and Fto null mice were grown for 24hr and harvested, after which endogenous FTO (left panel) or TRIP4 (right panel) proteins were immunoprecipitated using antibodies against FTO and TRIP4. Resulting immunoprecipitates were analysed by western blotting using indicated antibodies. (C) Schematic overview of the testing of the FTO binding epitopes that interact with TRIP4. Y2H experiments were performed to determine a direct interaction between full length TRIP4 and functional domain deletions of FTO. The results of this deletion mapping narrowed down the critical interacting domain of FTO to the region encoded by amino acids 30–48 and 249–269. The black lines mark the regions cloned into the yeast two-hybrid constructs and numbers in parentheses indicate amino acid number of each deletion mutants. The five-point “grading” scale was used to determine the strength of the binding: “++++” highest binding, “+++” high binding, “++” moderate binding, “+” low binding and “–“background binding. (D) Regulation of NFκB reporter activation by FTO-TRIP4 interactions were investigated using *Fto* null MEF cells transfected with a firefly luciferase NFκB reporter vector, along with the indicated expression constructs and a control plasmid expressing renilla luciferase. The expression constructs used include pcDNA3 expressing full length FTO (Flag-FTO), the C-terminal fragment of FTO containing sequence 201–505 (Flag-C'FTO) or the N-terminal fragment of FTO spanning aa 1–200 (Flag- N’FTO) with or without pcDNA3 expressing full length TRIP4. The firefly luciferase activity was normalized by renilla luciferase activity and expressed as fold change from control cells expressing only full-length FTO and the reporter construct.

We first showed in HEK cells transiently transfected with FLAG tagged FTO, that FLAG antibodies were able to co-immunoprecipitate TRIP4 (Figure 6A). Next, by using mouse embryonic fibroblasts (MEFs) obtained from WT mice, we demonstrated that immunoprecipitation of endogenous Fto could pull down endogenous *Trip4* (Figure 6B left panel) and *vice versa* (Figure 6B right panel). MEFs obtained from *Fto*^−/−^ mice were included as negative controls.

To confirm a direct interaction between FTO and TRIP4, we undertook an orthogonal approach using the widely used method for studying protein–protein interactions *in vivo*, the Yeast-2-hybrid (Y2H) system. By determining the interaction of full length human TRIP4 with different deletion mutants of FTO, we showed that amino acid residues 30–48 and 249–269 in FTO are the epitopes that directly interact with TRIP4 (Figure 6C). In the 3-dimensional structure, FTO binds to TRIP4 through two surface mobile epitopes exposed at one side of FTO (Supplemental Figure 8B).

To determine if FTO and TRIP4 functionally interact to influence the NFкB pathway, we used MEFs obtained from *Fto*^−/−^ mice and transiently transfected these with an NFкB-responsive luciferase reporter. To this we additionally transfected a TRIP4 expression construct together with a variety of constructs expressing tagged full-length FTO or deletion mutants. The MEFs expressing either FTO or TRIP4 individually, or expressing TRIP4 and N- or C-terminal FTO deletion mutants, did not show an increase in luciferase signal. However, when TRIP4 was expressed with either FLAG-tagged or Myc-tagged full length FTO, there was up to a five-fold increase in luciferase signal (Figure 6D). Thus, in the context of this cellular system, FTO appears to be necessary for TRIP4-dependent transactivation of NFкB.

## 4 Discussion

We report that mice deficient in Fto significantly increase their fat mass in response to HFD and that *Fto*^+/−^ and *Fto*^−/−^ mice remain sensitive to the anorexigenic effects of leptin, both after exposure to a HFD or after acute central application of palmitate. We show that genes encoding components of the NFкB signalling pathway are down-regulated in *Fto*-deficient mice following a HFD. Further, when this pathway is reactivated in *Fto*-deficient mice with a single low central dose of TNFα, the mice became less sensitive to the effects of leptin. We identify a transcriptional coactivator of NFкB, TRIP4, as a binding partner of FTO and a plausible candidate involved in mediating this phenomenon.

Our results are in contrast to the initial report by Fischer and colleagues [10], where losing just one functional copy of *Fto* was reported to protect animals from weight gain on a HFD. This difference could be due to the diet used in our study, which contained 60% of its calories from saturated fat, mostly derived from lard, whereas a diet with 42% of calories from milk-fat was used in the previous report. Further differences in the phenotype may be due to different gene targeting strategies, with Fischer et al. deleting exons two and three of *Fto*, whereas the model we report here only has exon 3 deleted.

We also report that despite a lowered fat mass in the *Fto* null mice, there was no difference in serum leptin levels between the three genotypes, either on regular chow or after a HFD. The determinants of circulating leptin levels are complex, not fully understood and not wholly determined by leptin sensitivity in the hypothalamus. At any given level of fat mass circulating leptin levels vary widely [26]. This is true even in conditions such as leptin receptor deficiency, where the cause of the leptin resistance is known [27]. Interestingly, transgenic mice carrying additional copies of *Fto*, in whom fat mass is increased, actually show relative hypoleptinemia [13] suggesting that *Fto* gene dosage may have an effect on leptin levels independent of its effect on fat mass.

Intriguingly, in spite of significant weight gain following the HFD, we show that losing just one functional copy of *Fto* was sufficient to ameliorate the leptin resistance and impaired glucose homeostasis normally associated with exposure to a HFD and an increase in fat mass. Previously, palmitoyl-coA has been shown to accumulate in the hypothalamus of animal models following a HFD [28] and central administration of palmitate leads to leptin resistance [17]. Mice that were completely or partially deficient in Fto were resistant to the effects of central palmitate infusion on leptin's action on food intake.

*Hypothalamic leptin resistance has been reported in both rats and mice within one to three days of exposure to HFD and prior to substantial weight gain* [29]*. In our study we showed that the loss of Fto ameliorates the development of central leptin resistance following a high-fat challenge. Of note, the HFD also engendered a differential distribution of fat between visceral and subcutaneous depots that mirrored the improved metabolic phenotype observed in FTO deficient mice* [30]*. This maybe as a result of a role for FTO in fat,* with *FTO* variants having been shown to be associated with both waist circumference and BMI [1], [31,32], as well as leading to a variation in the distribution of adipose tissue as determined by magnetic resonance imaging [33]; although *this has not been directly addressed here. Further, this differential adipocyte phenotype may also have, in part, impacted upon the persistent leptin sensitivity seen in the HFD fed Fto-deficient mice. However, given the fact that leptin sensitivity was also seen in the acute palmitate treated mice, we believe that the effect is due primarily, if not wholly, to central signalling of FTO. It is well-known that central leptin signalling can modulate glucose homeostasis* [34–36].

The concept of hypothalamic ‘microinflammation’, or more accurately the up-regulation of inflammatory markers within the medial basal hypothalamus, is now recognized as a potentially important process in the pathogenesis of HFD induced metabolic disorders [37]. For instance, there are data indicating high-fat feeding can influence leptin sensitivity, through the up-regulation of the *Tlr4* [21], *Myd88* [17], *Socs3* [38] and *Ptp1β* [39] in the hypothalamus which consequently impacts upon metabolic phenotypes [40,41]. Further, pharmacological or genetic disruption of the NFκB pathways have been shown to restore leptin sensitivity and reduce adiposity in diet induced obesity [18]. Consistent with these studies, we demonstrate here that the consumption of a HFD by WT mice causes an up-regulation of genes in the NFκB pathway in the hypothalamus. This response is significantly reduced with Fto-deficiency in a gene dose-dependent manner.

No difference in leptin sensitivity or NFκB signalling transcription profile was observed when mice were on standard chow, which is to be expected if NFκB is the plausible downstream mechanism for the resistance to leptin observed upon a HFD challenge. NFκB and its upstream regulator IKKb, are both expressed abundantly in the medial basal hypothalamus, remain suppressed under chow conditions and are activated by states of over-nutrition, including HFD and acute central overload of fatty acid and glucose [18]. This mirrors the nutritional regulation of Fto mRNA within the medial basal hypothalamus; being down-regulated by fasting [4,14,15] and up-regulated by feeding [42] and a HFD [15], [16].

A low dose of central administration of TNFα has been shown previously to be able to mimic some of the molecular changes seen within the hypothalamus after exposure to a HFD [21,23,43]. Our results show that the activation of NFκB signalling by low dose of TNFα restores the ability of palmitate to induce hypothalamic leptin resistance in Fto-deficiency, suggesting that Fto interacts with key molecules along the NFкB pathways to play a role in regulating central leptin sensitivity. Our data suggest that FTO could be a key molecular mediator of leptin resistance and lowering or inhibiting FTO activity may be a plausible therapeutic strategy to improving and/or preserving leptin sensitivity in obese states.

The domain composition of FTO in vertebrates and several algae pointed us towards TRIP4, a transcriptional co-activator of NFκB, as a possible mediator of this phenomenon. Our evidence of physical and functional interaction between FTO and TRIP4 suggests a possible mechanism linking FTO to NFκB signalling. Consequently, the up-regulation of Fto on a HFD appears to play a key role in the development of leptin resistance. Interestingly, the brain is the only organ that expresses high levels of both *Fto* and *Trip4*. Additionally, the co-ordinated down-regulation in NFκB gene expression between WT and Fto-deficient mice on a HFD is restricted to the hypothalamus, and is not observed in other brain regions or in the periphery. Our data also indicate that loss of Fto signalling specifically impairs HFD-induced NFκB activation while TNFα-induced NFκB activation appears to remain intact. Within the hypothalamus Fto is clearly not the sole activator of NFκB. Rather, our data indicate that FTO regulates NFκB gene expression specifically within the hypothalamus, with other brain regions and peripheral tissues unaffected. We hypothesize that the interaction with TRIP4 within the hypothalamus provides this specificity.

Implicating FTO as being able to functionally interact with the NFκB signalling pathway adds another role to an increasing list of putative functions for this molecule. Fto has been characterized as a demethylase with the ability to remove 3 methyl groups from single stranded nucleic acids [4]. More recent evidence indicates that Fto may also have a role in the sensing of amino acids [20], which potentially provides an explanation for the significant interaction observed between FTO genetic variation and dietary protein on appetite and food cravings [44]. How and if these mechanisms link back into the original observations of associating genomic variance around the *FTO* locus with human pathophysiology remains to be fully determined. Indeed, recent data focussing on obesity associated variants within *FTO* have implicated two neighbouring genes, *RPGRIP1L* and *IRX3*, as having a functional link between the SNP and the observed human phenotypes. As with *Fto*, perturbing the expression of these genes in mice results in a bodyweight phenotype, with homozygous deletion of *Irx3* resulting in a smaller mouse [7] while heterozygous deletion of *Rpgrip1l* leading to a mild obesity phenotype [8]. These studies have led to much discussion as to which of the genes close to the loci identified by the initial wave of GWAS are responsible, either wholly or partially, for association with a range of human phenotypic characteristics [45,46]. The most parsimonious explanation is that a number of genes in this region play an important role in determining body composition.

This emerging body of work illustrates the complex biology that can emerge from the deeper exploration of the basis for genomic variants robustly associated with human adiposity. Whatever the contribution of FTO itself to the influence of the polymorphisms within its first intron to human adiposity, it is clear that disturbance of the expression and function of FTO can influence the relationship between dietary composition and fat accumulation, and this supports the notion that pharmacological modulation of the activity of FTO might have the potential for therapeutic benefit in a manner that is influenced by the nutritional environment.

## Disclosure statement

The authors have nothing to disclose.

## Figures and Tables

**Figure:**
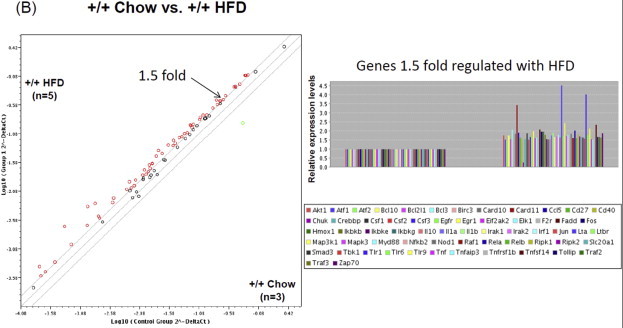


## Appendix A Supplementary data

Supplemental Table 1 All genes involved in the NFκB pathway that are presented on the NFκB signalling pathway profiler PCR array. Fold changes in expression in the wild type mice (HFD, n = 5) following 5 weeks of 60% HFD are compared to the wild type control mice on standard chow (chow, n = 3) and the directions of change are indicted by either ‘up’ or ‘down’.

Supplemental Table 2 All genes involved in the NFκB pathways that are presented on the NFκB signalling pathway profiler PCR arrays. Fold changes in expression in the *Fto*^+/−^ (+/−) or *Fto*^−/−^ (−/−) are compared to wild type controls following 5 weeks of 60% HFD and the directions of change are indicted by either ‘up’ or ‘down’ (n = 5 mice per group).
